# A single-cell micro-trench platform for automatic monitoring of cell division and apoptosis after chemotherapeutic drug administration

**DOI:** 10.1038/s41598-018-36508-8

**Published:** 2018-12-21

**Authors:** E. I. Chatzopoulou, P. Raharja-Liu, A. Murschhauser, F. Sekhavati, F. Buggenthin, A. M. Vollmar, C. Marr, J. O. Rädler

**Affiliations:** 1 Faculty of Physics, Ludwig- Maximilians-Universität, Munich, Germany; 2 Institute of Computational Biology, Helmholtz Zentrum München–German Research Center for Environmental Health, Neuherberg, Germany; 3 Department of Pharmacy, Ludwig-Maximilians-Universität, Munich, Germany

## Abstract

Cells vary in their dynamic response to external stimuli, due to stochastic fluctuations and non-uniform progression through the cell cycle. Hence, single-cell studies are required to reveal the range of heterogeneity in their responses to defined perturbations, which provides detailed insight into signaling processes. Here, we present a time-lapse study using arrays of micro-trenches to monitor the timing of cell division and apoptosis in non-adherent cells at the single-cell level. By employing automated cell tracking and division detection, we precisely determine cell cycle duration and sister-cell correlations for hundreds of individual cells in parallel. As a model application we study the response of leukemia cells to the chemostatic drug vincristine as a function of cell cycle phase. The time-to-death after drug addition is found to depend both on drug concentration and cell cycle phase. The resulting timing and dose-response distributions were reproduced in control experiments using synchronized cell populations. Interestingly, in non-synchronized cells, the time-to-death intervals for sister cells appear to be correlated. Our study demonstrates the practical benefits of micro-trench arrays as a platform for high-throughput, single-cell time-lapse studies on cell cycle dependence, correlations and cell fate decisions in general.

## Introduction

Cell-to-cell variability in responses to external stimuli is a pervasive feature of cellular systems, which prevails even in isogenic cell populations^1,2^. Such heterogeneity can be caused by epigenetic modifications, differences in cell cycle phase, or stochastic variations in gene expression and metabolic state. To dissect the sources of heterogeneity, the contextual role of cell cycle timing in the response to the stimulus needs to be investigated. Ideally, responses should be monitored in single cells over time to avoid the typical averaging effect intrinsic to population measurements. Time-lapse imaging has often been employed for this purpose, since it allows one to record cell divisions, track the fates of individual cells and reveal genealogical relationships^3–5^. To study the effect of cell cycle phase on stimulus response with high statistical power, large numbers of single cells must be observed continuously.

Tracking of cells, especially of non-adherent cultures, constitutes the typical bottleneck in implementing high-throughput time-lapse microscopy analyses. Various tracking algorithms have been proposed and evaluated^6,7^, but for practical purposes, the crucial parameter is the ratio of the time required to manually track single cells to the workload involved in correcting erroneous automated tracks^8^. For long-term tracking of fast-moving cells at high cell densities, efficient manual tracking is often the method of choice^9,10^. Spatial confinement of cells reduces the incidence of tracking errors and facilitates the application of tracking algorithms. Among the techniques available for capturing non-adherent cells for long-term observation, microfluidic devices^11^ as well as microwell platforms^12–20^ have been developed. Micro-fabricated arrays that sequester proliferating single cells and thus lead to spatially separated progeny clones serve as an especially useful tool for high-throughput investigations of cell cycle length, sister-cell correlations, and the impact of cell cycle phase differences on cell-to-cell variability.

The implications of cell-to-cell heterogeneity are of paramount importance for cancer progression and treatment^21^. Tumors of all types not only exhibit genetic diversity, they also display in response kinetics when exposed to chemotherapy^22–24^. Most state-of-the-art chemotherapeutic agents in clinical use target rapidly dividing cells and trigger apoptosis. Thus, vincristine, an antitumor vinca alkaloid, binds to tubulin and blocks chromosome segregation during metaphase^25,26^. In contrast, daunorubicin, an anthracycline aminoglycoside antineoplastic, intercalates into DNA and inhibits the function of the enzyme topoisomerase II during transcription and replication^27^. Both drugs are routinely used to treat a number of neoplasms^28,29^, including acute myeloid leukemia (AML)^30,31^. Yet, their exact effects on the timing of apoptosis at the single-cell level have not yet been explored.

Here, we introduce arrays of micro-trenches that facilitate continuous observation of individual, non-adherent cells. We demonstrate that automated image analysis using automated cell tracking permits precise determination of the distribution of cell cycle duration and detection of sister-cell correlations. We then study the time-to-death dynamics after administration of vincristine or daunorubicin, and compare the responses of chemically synchronized and non-synchronized populations. We find that, in the presence of the anti-mitotic agent vincristine, the time-to-death interval decreases as the cell cycle progresses. In contrast, no such effect is observed in the case of the topoisomerase II inhibitor daunorubicin. These results are consistent with experiments using cells that were synchronized using standard thymidine cell cycle arrest. Moreover, we find the time-to-death of sister cells to be strongly correlated in the unsynchronized population.

## Results

### The single-cell micro-trench platform

**Figure 1:**
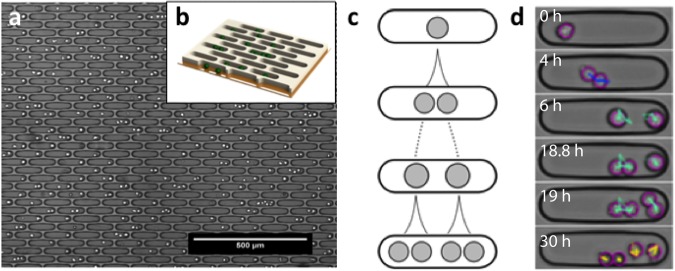
The micro-trench array enables long-term observation of single cell lineages. (**a**) Phase-contrast microscopy image of micro-trenches loaded with cells. (**b**) Schematic 3D representation of the 20 μm deep micro-trenches. The green spheres represent non-adherent cells. (**c**) Schematic diagram of cell division events within a single micro-trench. (**d**) Single cells can be automatically identified (purple circles around the cells) and followed over time in the time-lapse phase-contrast images (blue, cyan and yellow trajectories) of single micro-trenches. The trajectories in each frame show the last 10 steps. The length of the micro-trench is 120 μm.

To facilitate tracking of non-adherent cells over several generations in a label-free manner, we designed arrays of micro-trenches made of the biocompatible hydrogel polyethylene(glycol) diacrylate (PEG-DA; the fabrication of these arrays is described in Materials and Methods). The trenches are 30 μm wide, 120 μm long and 20 μm deep and can accommodate up to six non-adherent cells of the human leukemia cell line MOLM-13 **(**Fig. 1a,b**)**, used here as a cancer model system. The ratio of the width of a micro-trench to the diameter of a cell is around 2. Time-lapse experiments were carried out for up to 40 h, which is sufficient to observe two consecutive cell divisions and hence the first and second generations of daughter cells derived from the single starting cell per trench **(**Fig. 1c**)**.

Error-free tracking of single cells in time-lapse experiments is complicated by cell movement, interaction of cells, different fields of view, and image quality^8^. Use of micro-trench arrays simplifies the task considerably by (i) compartmentalizing the cell population and allowing one to monitor single cell lineages, (ii) avoiding interactions between the progenies of different starting cells, thus preventing errors due to mixed lineages, and (iii) reducing the problem of tracking thousands of cells simultaneously to many small, trench-specific tasks, which speeds up computation. To track the cells in an automated fashion (see Materials and Methods) we acquired slightly defocused phase-contrast images (focus at 20 μm below focal plane), which results in images with slightly blurred but clearly peaked intensity distributions^32,33^. In this study, we automatically track single starting cells in micro-trenches then filter out unreliable tracks and analyze cell cycle times and drug responses in more than 2700 single cells in two experiments (Tables 1 and 2). Figure 1d shows representative tracks of a single starting cell that undergoes two consecutive divisions.

**Table 1:** Cells analyzed in the unsynchronized population.

Detected micro-trenches in total	13994
Cells detected in micro-trenches at *t* = 0	3306
Micro-trenches with a single starting cell at *t* = 0	2765
Micro-trenches with two starting cells at *t* = 0	465
Micro-trenches with three starting cells at *t* = 0	63
Single starting cells that divide before drug addition	1443
Single starting cells that divided once before drug addition	442
Genealogies with one division before drug addition and apoptotic cells	172

**Table 2:** Cells analyzed in the synchronized population.

Detected micro-trenches in total	13428
Cells detected in micro-trenches at *t* = 0	2546
Micro-trenches containing a single cell at *t* = 0	2224
Micro-trenches containing two cells at *t* = 0	287
Micro-trenches containing three cells at *t* = 0	35
Micro-trenches with single cells during measurement	1927

### Distribution of cell cycle duration in single cell lineages

**Figure 2:**
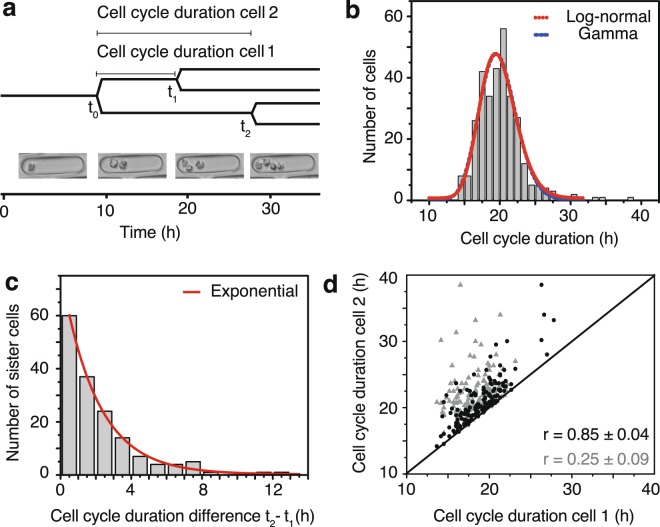
Micro-trenches enable precise determination of the distribution of cell cycle durations. (**a**) Schematic representation of cell division events. The axis at the bottom indicates the time (in h) and shows representative phase-contrast images at selected time-points. (**b**) Measured distribution of the cell cycle duration for an ensemble of 320 cells (MOLM-13) with a mean of 19.7 h and standard deviation of 2.6 h. The dotted red line corresponds to a log-normal fit and the dashed blue line to a gamma distribution fit. (**c**) Distribution of the difference between the cell cycle durations for sister-cell pairs. Differences in cell cycle duration are fitted with an exponential curve (red line). (**d**) The cell cycle durations of sister cells (black dots) show higher correlation (Pearson correlation coefficient *r* = 0.85 ± 0.04) than those of randomly paired cells (grey triangles, *r* = 0.25 ± 0.09).

After the first division of a single starting cell, a micro-trench contains two daughter cells that can be separately tracked **(**Fig. 2**)**. For each cell, we determine the first division time point t_0_, the division of the first daughter cell at time t_1_, and the division of the second daughter cell at t_2_ (Fig. 2a**)**. In our experiments 320 MOLM-13 starting cells were observed for 40 h through at least two divisions. The distribution of cell cycle durations, with a mean of 19.7 ± 2.6 h (mean ± std, n = 320 cells) is well described by both a log-normal distribution and a gamma distribution (Fig. 2b). For clones in which both t_1_ and t_2_ were observed (n = 320), we analyzed the difference between the cell cycle durations for sister cells, t_1_ – t_2_. The distribution of sister-cell differences has a mean of 2.3 ± 2.7 h (n = 85 pairs of sister cells) and is well fitted with an exponential distribution (Fig. 2c). Compared to randomly paired cells, the cell cycle durations of sister cells are highly correlated, with a Pearson correlation coefficient of *r* = 0.85 ± 0.04, as compared to *r* = 0.25 ± 0.09 for randomly paired cells.

### Case study: Vincristine/daunorubicin-induced apoptosis

**Figure 3:**
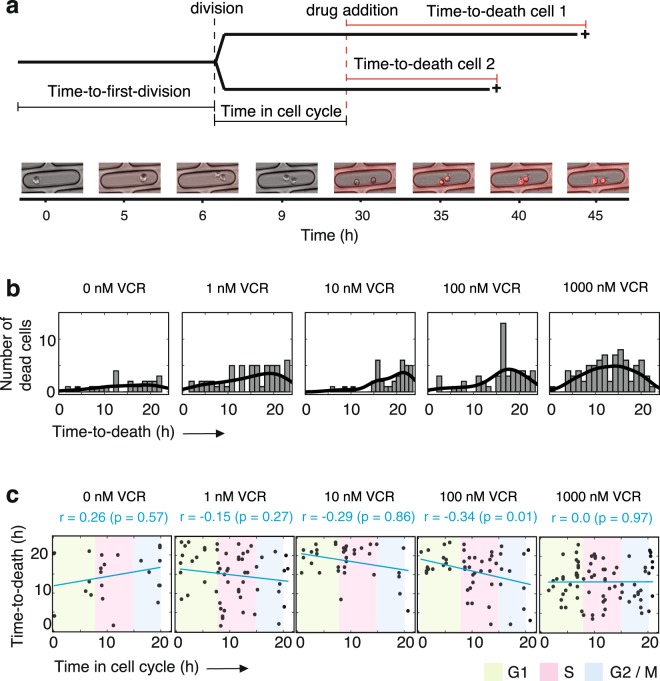
Vincristine induced time-to-death in an unsynchronized cell population is drug concentration dependent. (**a**) Schematic description of the experimental procedure. Image acquisition starts at time 0 h and after a defined time (20 h), vincristine is added. Tracking of the individual cells reveals the time at which each mother cell divides, which is different in each micro-trench, and the time-to-death of the two daughter cells. Images show a representative cell that has divided before drug addition; both of its daughter cells die (overlay of the in-focus phase-contrast and PI fluorescence images). (**b**) Distribution of the time-to-death for all tracked cells. The black lines represent the kernel density estimation of the probability density function for each drug treatment. (**c**) Correlation plots between the time passed in (i.e., extent of progression through) the cell cycle and the time-to-death. The blue line is a linear fit to the scatter plot for each drug treatment. The Pearson correlation coefficient (*r*) for each drug concentration is shown above each graph, together with the p-value of the correlation test (in parenthesis). The colored areas denote the different cell cycle phases, based on the average division time presented in Fig. 2b. Green stands for the G1, pink for the S and blue for the G2/M phase.

It is generally accepted that regulation of the cell cycle is perturbed in cancer. Chemotherapeutic drugs can have either a cytostatic or cytotoxic effect, depending on the phase of the cell cycle at which they first encounter their target cells^34^. Here, we utilize our micro-trench set-up to monitor cell cycle progression based on phase-contrast images, and the time-to-death from fluorescence image analysis. We test whether the activity of two widely used chemotherapeutic drugs, vincristine and daunorubicin, is affected by the phase of the cell cycle of MOLM-13 cells. The first division of the starting cell t_0_ is used as a reference point. Based on the previously measured cell cycle duration distribution (Fig. 2b**)** each drug was added after 20 h, when most starting cells had divided once (Fig. 3a). Phase-contrast images were taken every 10 min and fluorescence images every 30 min. Cell death was assessed via a detectable PI fluorescence (see Materials and Methods). An exemplary movie of cells dividing and undergoing apoptosis after addition of vincristine can be seen in Supplementary Movie 1.

The measured time-to-death distribution, i.e. the time elapsed between the addition of the drug and the death of the cell is shown in Fig. 3b for increasing drug concentrations. As expected, only a few cells die under control conditions, when no drug is added, while the number of dead cells rises with increasing vincristine concentration. At 10 and 100 nM vincristine, the number of dead cells increases considerably after 12 hours of exposure, indicating a time scale for the delay between drug addition and cell death. The scatter plots in Fig. 3c, for each drug treatment, correlate the stage in the cell cycle at which the cell first encounters the drug (where the duration for each phase is calculated based on the phase durations proposed in^35^) with the time-to-death. While in the control the correlation is slightly positive, with increasing vincristine concentration the correlation becomes increasingly negative. At the highest concentration, 1000 nM, no correlation is observed, indicating that side-effect toxicities are prominent. The negative correlation between the extent of progression through the cell cycle and the time-to-death in the case of vincristine is expected, since it is known that at high concentration, vincristine stimulates microtubule depolymerization and mitotic spindle destruction, while at lower, clinically relevant concentrations, it blocks mitotic progression^36^. Hence, the longer the time elapsed since a cell’s previous division, the closer it should be to the M-phase, and should therefore have a shorter time-to-death. In the case of daunorubicin (10 and 100 nM), no correlation between the time since the previous division and the time-to-death was observed (Supplementary Fig. 3b).

**Figure 4:**
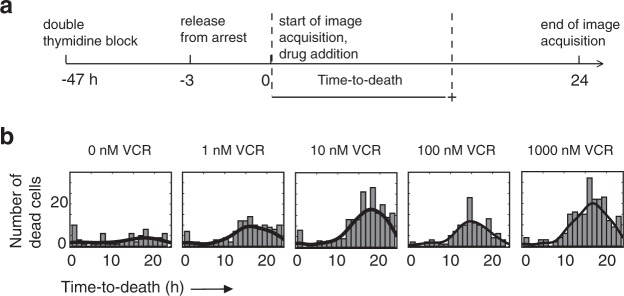
Synchronized cell population: distributions of the time-to-death in the presence of vincristine. (**a**) Timeline of the experimental procedure. (**b**) Here, the time-to-death of all cells is analyzed, irrespective of whether they divided after drug addition or not. For each drug concentration, the plots show the distribution of the time-to-death for synchronized cells. The black lines represent the kernel density estimation of the probability density function in each case.

To investigate the role of cell cycle phases further, we used our pipeline with a cell population that had been synchronized with the “double thymidine block” procedure^37^ using micro-trenches (Fig. 4). Thymidine block arrests cells at the transition between G1 and S, just prior to the initiation of DNA replication. Cells were released 3 h from the block before the start of imaging and seeded in micro-trenches, and the drug was added just before the onset of image acquisition (Fig. 4a**)**. For all vincristine concentrations, the peak of the time-to-death distribution **(**Fig. 4b**)** occurs more than 12 h after starting imaging, similar to that observed in the unsynchronized population **(**Fig. 3b**)**. The time-to-death distributions associated with daunorubicin exposure are similar for both unsynchronized and synchronized cells (Supplementary Figs 3a, 4a,b).

**Figure 5:**
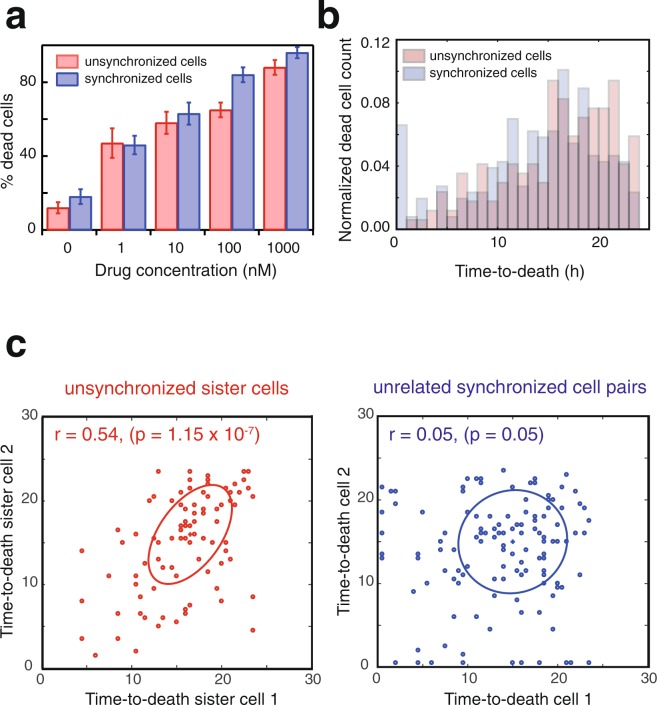
Vincristine induces correlated times-to-death in sister cells. (**a**) Dose response curve, i.e. percentage of dead cells versus drug concentration for unsynchronized and synchronized cells. Error bars were calculated to reflect the 95% interval. (**b**) Distributions of time-to-death of the normalized number of cells across all drug concentrations for both the unsynchronized and synchronized populations. Note that synchronization does not affect the time-to-death distribution. (**c**) Sister cell pairs derived from the unsynchronized population (n = 93 pairs of sister cells) exhibit similar times-to-death (*r* = 0.54, p-value: 1.15 × 10^−7^), while for unrelated cells in the synchronized population (n = 129 pairs) no correlation is observed (*r* = 0.05, p-value: 0.05). The ellipses indicate the directionality of the correlation.

For both, the unsynchronized and synchronized populations, the total number of dead cells as a function of vincristine concentration is similar (Fig. 5a). The IC50 value derived from these data for the unsynchronized population is 652 ± 90 nM, while for the synchronized cells is 9 ± 2 nM. Thus, we observe that synchronized cells are more sensitive to the vincristine treatment. Whether a drug is more potent against synchronized cells is context specific. Previously it has been reported that cell cycle arrested HeLa cells become resistant to doxorubicin and cisplatin treatment^38^. On the other hand, cell cycle arrest increased TRAIL-induced apoptosis^39^. Also the time-to-death distributions are similar for both populations (Fig. 5b). However, while in the unsynchronized population, the time-to-death correlation between sister cells in the same micro-trench is evident (Fig. 5c, Pearson correlation coefficient *r* = 0.54, p-value 1.15 × 10^−7^), we see no correlation between unrelated cells (*r* = 0.05, p-value 0.05) in the synchronized population experiment.

## Discussion

The data presented above show that arrays of micro-trenches facilitate automated tracking of cells in parallel and allow one to deduce the lengths of individual cell cycles without the use of cell cycle-specific molecular markers. Using automated image analysis, we determined the time of the first cell division in each trench, which provides a reference point for the initiation of each ensuing cell lineage. Compartmentalization of a cell population into small groups enables time-lapse analysis of both adherent and non-adherent cells over periods exceeding 48 h. When plated on a plain surface, cells quickly escape from the field of view and misidentification of adjacent cells leads to errors, such that extensive computational power and time is required to ensure single-cell tracking. We observed that the distribution of cell cycle durations in non-synchronized mammalian cells is well fitted by a log-normal distribution; this also holds for cell size as reported previously^40,41^. Moreover, in agreement with a previous study^42^, we found cell cycle durations to be correlated between sister cells.

We have demonstrated the practicability of clinically relevant, time-resolved single-cell studies in the micro-trench platform by measuring the time-to-death after the addition of the cancer drugs vincristine and daunorubicin. We found that the time-to-death in the presence of vincristine is negatively correlated with the extent of progression through the cell cycle at the time of its administration, and that this correlation becomes more prominent with increasing vincristine concentration up to 100 nM. However, there is no correlation at the highest concentration of vincristine (1000 nM). Phase-independent apoptosis induced by microtubule-targeting agents has been reported in previous studies utilizing other cell lines and relatively high drug concentrations^25,43,44^, which suggests that the lack of correlation at 1000 nM vincristine might be due to the accumulation of side-effects over the course of the whole cell cycle. It has been shown that, after exposure to antimitotic drugs, cells display complex fate profiles, ranging from unequal cell divisions that generate aneuploid daughter cells to exit from the cell cycle without undergoing cell division (mitotic slippage), and leaving G1 and undergoing apoptosis or senescence^3^. We furthermore found that the time-to-death is correlated in sister-cell pairs derived from unsynchronized populations. Variability in sister-cell responses is a striking phenomenon, since it provides hints as to whether different phenotypes stem from genetic differences or adventitious differences in the compositions of cellular proteomes. It has previously been reported that sister cells tend to undergo apoptosis quite synchronously^45,46^. However, in the presence of antimitotic drugs, the fate of one sister was found to be independent of the fate of the other^3^. In another study, the response to TRAIL-induced apoptosis was correlated between sister cells, as was the time-to-death, although the latter correlation decayed as a function of time within the 8 h observation window^47^. All these observations, as well as those reported here, are possibly explained by transient heritability, a model which assumes that fluctuations in protein synthesis promote cell phenotype divergence^47^.

Time-resolved studies will be instrumental in scrutinizing the time dependence of molecular determinants within cellular decision-making networks. The investigation of chemotherapeutic drug dynamics in particular can be extended to the application of multiple drugs, as used in combination therapy, in order to explore the effect of time-delayed applications and the optimal timing for the administration of combinations of drugs. For these investigations with potentially subtle effects on the single-cell level, a large number of measured cells are required. To this end, micro-trenches combined with time-lapse microscopy and automated image analysis represent a methodological advance, which enables versatile high-throughput long-term observations with large statistical power.

## Materials and Methods

### Cell culture

The acute monocytic leukemia (AML-M5a) cell line MOLM-13 was cultured in RPMI 1640 GlutaMAX medium (Gibco®) supplemented with 20% (vol/vol) Fetal Bovine Serum (FBS, Gibco®), both purchased from Life Technologies GmbH, Darmstadt, Germany

### Fabrication of micro-trench arrays

#### Photolithography of the SU-8 wafer

The SU-8 (MicroChem Corp, USA) wafer was fabricated in an in-house cleanroom facility using a ProtoLaser LDI system (LPKF Laser & Electronika, Naklo, Slovenia) with a 375 nm wavelength laser and 1 μm spot diameter.

#### Soft lithography and micromolding

Polydimethylsiloxane (PDMS) prepolymer solution was mixed with the cross-linker in a 10:1 ratio (w/w) (Sylgard 184, Dow Corning, USA) and then degassed under vacuum. PDMS was then poured onto the SU-8 wafer, degassed and cured at 50 °C. The resulting PDMS stamp was then peeled off the wafer and cut into appropriate shapes. The PDMS pieces, patterned with pillars 20 μm high, were activated with argon plasma and immediately placed upside down on a silanized TMSPMA (3-(trimethoxysilyl)propyl methacrylate, Sigma-Aldrich, Germany) glass coverslip. A drop of freshly prepared solution of PEG-DA (Mn = 258) containing 2% (v/v) 2-hydroxy-2-methylpropiophenone (both from Sigma-Aldrich, Germany) was placed at the edge of the PDMS stamp, which fills it by capillary flow. PEG-DA is then polymerized in an UV-ozone cleaning system (UVOH 150 LAB, FHR, Ottendorf, Germany). Next, the PDMS stamps were peeled off and the resulting micro-trenches of cross-linked PEG-DA are dried in an oven (Binder GmbH, Tuttlingen, Germany) overnight at 50 °C. Finally, the slides were sonicated in the presence of 70% ethanol in distilled water before a sticky slide is attached on top (8-well sticky slide, ibidi GmbH, Munich, Germany). The ibidi® 8-well slide compartmentalizes the glass slide in 8 different parts. The advantage is that 8 different conditions, in our case 8 different drug concentrations, can be monitored simultaneously. Each compartment contains about 2500 micro-trenches, which cover about the 1/3 of the available surface of the compartment. However, to keep the time resolution at an acceptable level, namely 10 minutes, we monitored on average 1750 micro-trenches per condition. This protocol is based on a method previously described^48^.

### Time-lapse fluorescence microscopy

#### Sample preparation

Freshly prepared slides, each bearing about 20,000 micro-trenches, were sterilized with 80% ethanol for 2 h and then coated with a (35 μg/mL) fibronectin solution (YO Proteins, Huddinge, Sweden) for 45 min. The cell medium used during the measurements was RMPI 1640 (without phenol red) supplemented with 20% (v/v) FBS, 2 mM GlutaMAX, penicillin and streptomycin (100 units/mL and 100 μg/mL respectively), and 1 mM sodium pyruvate (all from Life Technologies GmbH, Darmstadt, Germany). Cells were seeded in the wells at a low concentration (45,000 cells/mL), to achieve single-cell occupation in each micro-trench. To detect dead and apoptotic cells, propidium iodide (PI; Novus Biologicals, Littleton, USA) and Cell Event™ Caspase 3/7 (ThermoFischer Scientific, MA, USA) markers were used at 5 μl/mL and 80 μl/mL, respectively. Since daunorubicin is autofluorescent in the red region, the PI marker was omitted in that case.

Imaging was performed with an inverted Nikon Ti Eclipse microscope equipped with a motorized stage (Tango XY Stage Controller, Märzhäuser Wetzlar GmbH & Co. KG, Germany), a CFI Plan Fluor DL 10X objective, a pco.edge 4.2 camera (PCO AG, Kelheim, Germany) and a Lumencor Spectra LED fluorescence lamp. For detection of the caspase 3/7 and the PI marker, the following filters were used respectively, 474/27 nm, 554/23 (excitation) and 515/35 nm, 595/35 nm (emission). Defocused (−20 μm) phase-contrast images were taken every 10 min and in-focus phase-contrast and fluorescence images were acquired every 30 minutes for 48 hours. Vincristine or daunorubicin was added 20 h after the beginning of the imaging. During the recording, samples were kept at a constant temperature of 37 °C and CO_2_ concentration using an Okolab heating and CO_2_ box (OKOLAB S.R.L., NA, Italy). To synchronize the cell population used for comparative purposes, the double thymidine block protocol was followed. Briefly, MOLM-13 cells in the exponential growth phase were incubated in blocking medium (culture medium supplemented with 2 mM thymidine (CAS 50-89-5, Calbiochem®, Germany)) for 24 h. Cells were then released from the block, incubated in culture medium for 8 h and finally in blocking medium for 12 h. After 2 h, the synchronized population was seeded in the slide bearing the micro-trenches, together with the markers and drugs under the conditions used for the unsynchronized population, and imaged for 24 h.

### Image processing and data analysis

#### Tracking in the phase-contrast channel

Each out-of-focus phase-contrast image was processed, using the Jython plugin in Fiji, as follows. First, a Gaussian blur correction^49^ is applied followed by local contrast normalization and minimum error thresholding^50^. To reduce static noise (such as micro-trench margins), a mean correction is applied by subtracting from each slice a time-independent mean intensity averaged over all slices. This creates a mask that is subtracted from the phase-contrast image, and single cells are identified using the Laplacian of Gaussian detection method. The detected cells are concatenated into tracks and cell trees using the Linear Assignment Problem^51^ in the TrackMate plug-in in Fiji. The image analysis pipeline is visualized in Supplementary Fig. S1. The following equation describes the cells successfully tracked automatically in the phase-contrast channel: N_0_ = N_t_ + N_not divided_ + N_losses_ here, N_0_ is the number of single starting cells at the beginning of the measurement (t = 0), N_t_ the number of cells that were tracked and underwent division, N_not divided_ is the number of cells that did not divide but were successfully tracked by the algorithm, and N_losses_ is the number of cells that the algorithm lost. With our approach, we were able to track 50% of the whole cell population tested.

#### Tracking in the fluorescent channel

Fluorescence images are used to detect cell death. Unlike the defocused phase-contrast channel, these images undergo only brightness and contrast adjustment, followed by mean correction of static noise as described above. The rest of our image computing pipeline is identical (see Fig. S2).

#### Slit assignment of tracks

To assign tracked clones to micro-trenches, the mask of the trench array is used to associate tracks imaged by both phase-contrast and fluorescence. Each contour in the mask image (seen as black rod-like forms) is assigned a unique identity, which is then used as the association identifier for tracks in both phase-contrast and fluorescent data (see Fig. S2). The time of cell death is determined from the onset of the PI or caspase 3/7 signal.

#### Estimating time-to-death using phase-contrast and fluorescence tracking data

The fluorescence tracks were used to determine the time-to-death. When a fluorescent cell track was detected in the same micro-trench as a phase-contrast cell track, the first measured time point of the fluorescent track was used to calculate time-to-death.

## Electronic supplementary material

Supplementary Information

## Data Availability

The image and tracking data was combined and post-processed using Python scripts utilizing OpenCV, NumPy, pandas and Matplotlib. Our code is available at: https://github.com/raharjaliu/microtrench-chemotherapeutic-vision.
