# Supplementary material for: A single-cell micro-trench platform for automatic monitoring of cell division and apoptosis after chemotherapeutic drug administration
Source: Sci Rep. 2018 Dec 21;8:18042. doi: 10.1038/s41598-018-36508-8 (PMC6303304; doi:10.1038/s41598-018-36508-8)
Supplement: Supplementary file 1 — Supplementary Information [file 41598_2018_36508_MOESM1_ESM.docx]

**Supplementary Information**

**A single-cell micro trench platform for automatic monitoring of cell division and apoptosis after chemotherapeutic drug administration**

E.I. Chatzopoulou^1^, P. Raharja-Liu^2^, A. Murschhauser^1^, F. Sekhavati^1^, F. Buggenthin^2^, A. M. Vollmar^3^, C. Marr^2, #^ and J.O. Rädler^1, #^

^1^ Faculty of Physics Ludwig- Maximilians-Universität Munich Germany

^2^ Institute of Computational Biology Helmholtz Zentrum München–German Research Center for Environmental Health Neuherberg Germany

^3^ Department of Pharmacy Ludwig-Maximilians-Universität Munich Germany


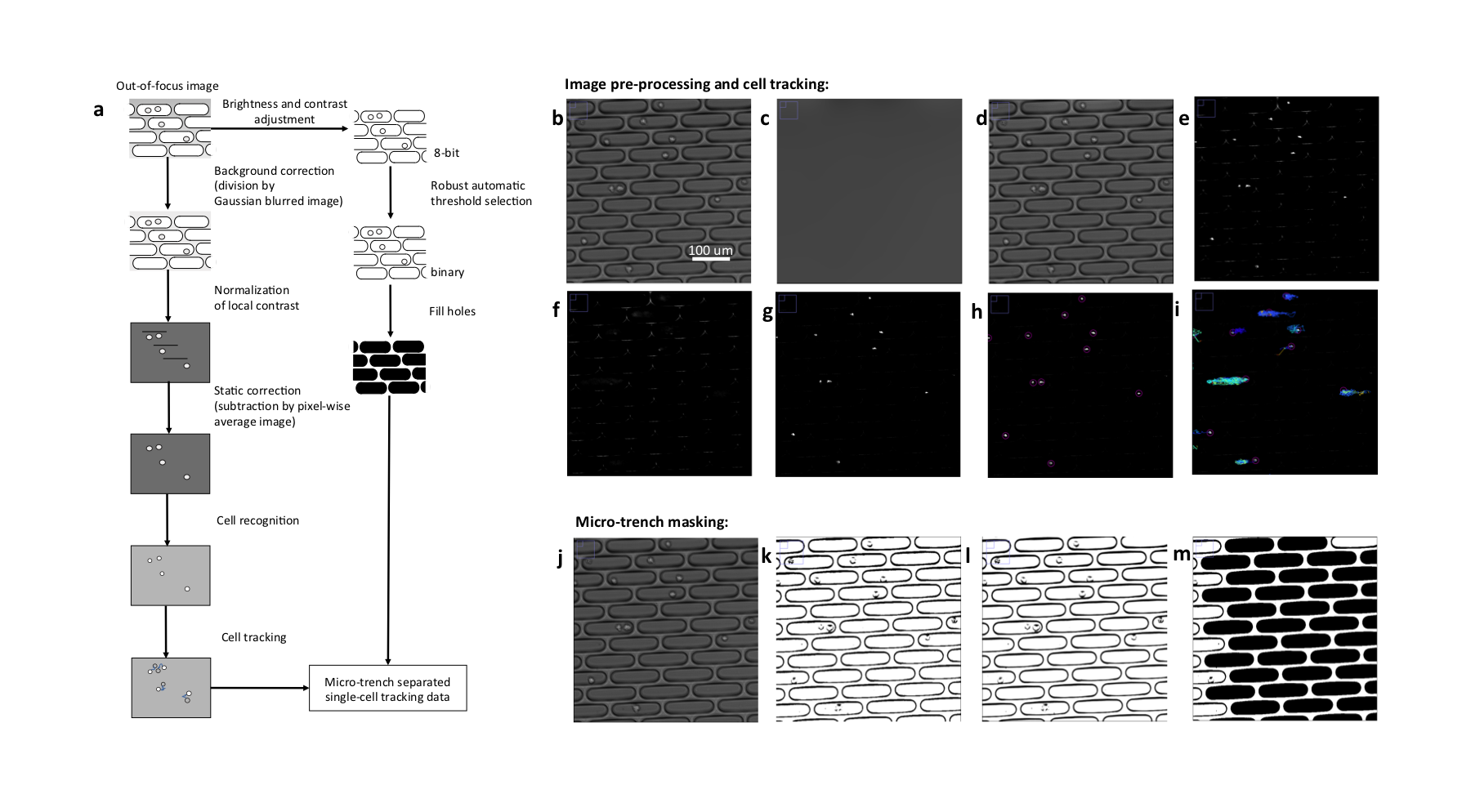


**Supplementary Figure S1: Image analysis pipeline:** (a) The image processing pipeline in the phase contrast channel. (b) The input image. (c) The result of Gaussian blur on input image. (d) The input image after correction with Gaussian blur. (e) The output of the local contrast normalization on (d). (f) The pixel-wise mean intensity image of (e). (g) The output of the static correction (subtraction of pixel-wise average). (h) Detected cells in the image (g). (i) The cell tracks superimposed on the image (g). (j) The same input image as in (b). (k) The input image after brightness and contrast adjustment. (l) The output of robust automatic threshold selection (RATS) on image (k). (m) The output of the holes filling algorithm.

**
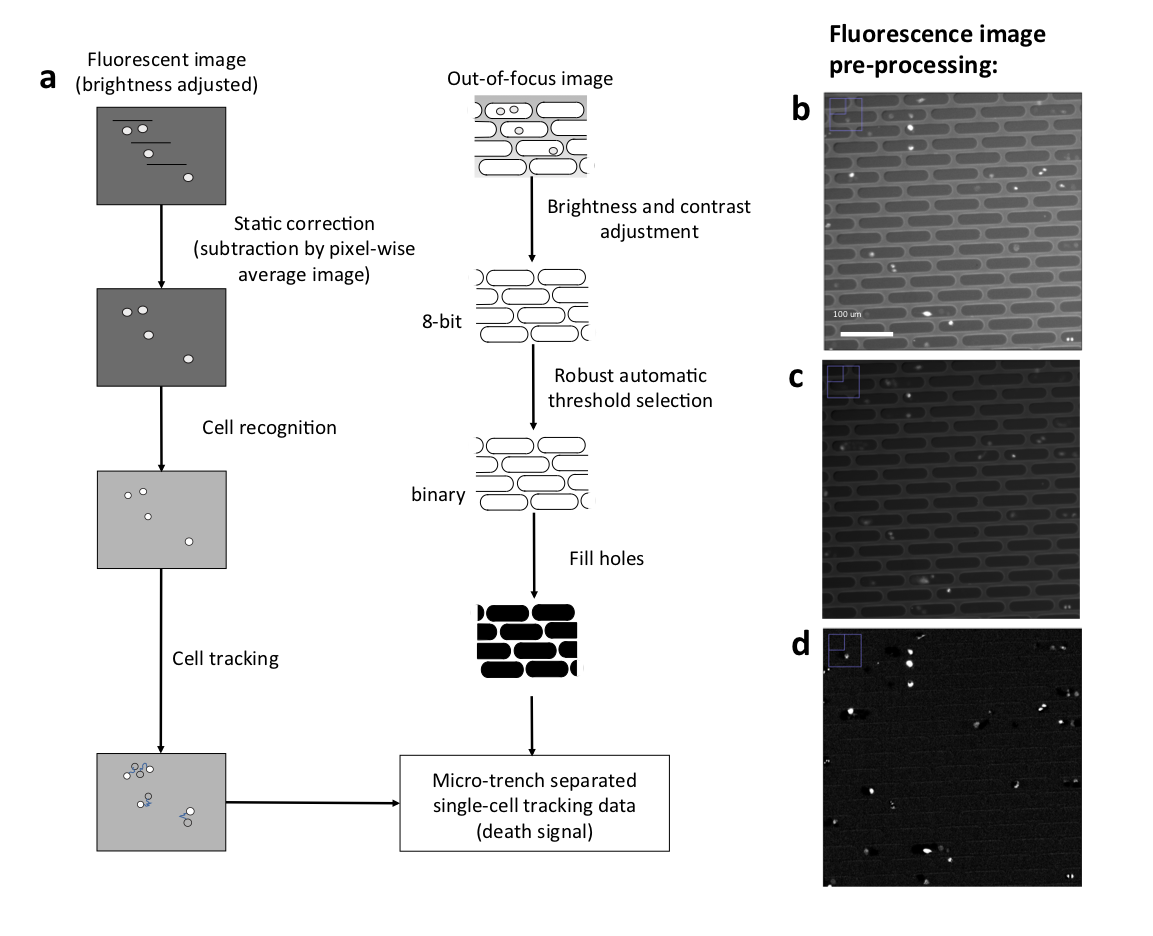
**

**Supplementary Figure S2:** (a) The image processing pipeline in the fluorescence channel. (b) An input image. (c) The input image after adjusting for the brightness and contrast. (d) The pixel-wise mean correction on (c).

**
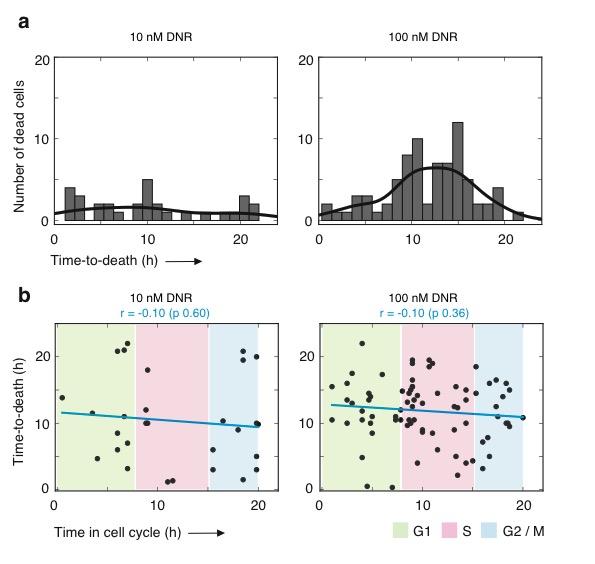
**

**Supplementary Figure S3: Unsynchronized cell population: concentration dependent effects on the time-to-death induced by daunorubicin.** (a) Distribution of the time-to-death for all tracked cells. The black lines represent the Kernel density estimation of the probability density function in each drug treatment. The percentage of dead cells is 47 ± 5 % in the case of 10 nM and 94 ± 3 % for 100 nM. (b) No correlation is observed between the time passed in the cell cycle and the time-to-death. The blue line is a linear fit to the scatter plot in each drug treatment. The Pearson correlation coefficient (r) is shown for each drug concentration on top of each graph together with the p-value of the correlation test (in parenthesis). The colored areas denote the different cell cycle phases based on the average division time presented in Figure 2b. Green stands for the G1, pink for the S and blue for the G2/M phase.


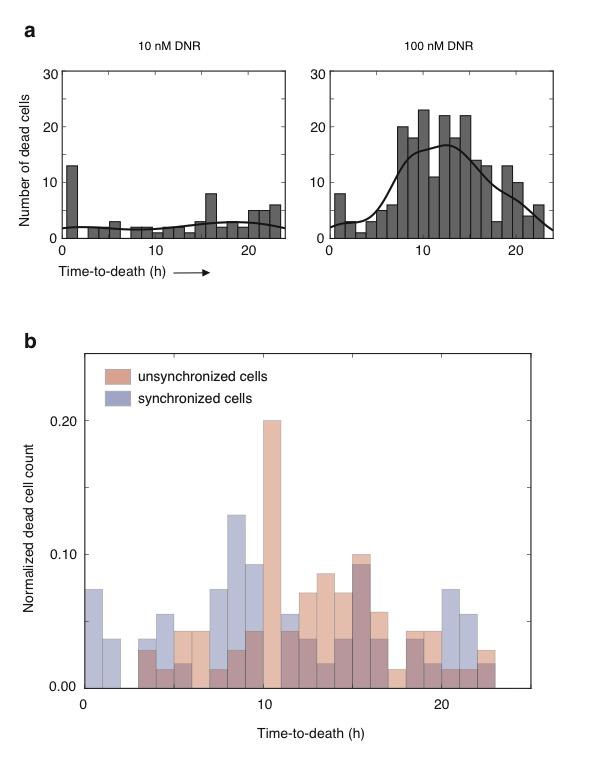


**Supplementary Figure S4: Synchronized cell population: distributions of the time-to-death induced by Daunorubicin.** (a) The time-to-death of all cells is analyzed, whether they divided after drug addition or not. For each drug concentration, the plots show the time-to-death distribution of synchronized cells. The black lines represent the Kernel density estimation of the probability density function in each case. The percentage of dead cells is 52 ± 5 % in the case of 10 nM and 77 ± 6 % for 100 nM. (b) The synchronization protocol does not affect the time-to-death; the shape of both distributions is equivalent.
